# The Central Role of the Inflammatory Response in Understanding the Heterogeneity of Sepsis-3

**DOI:** 10.1155/2018/5086516

**Published:** 2018-06-07

**Authors:** Renyu Ding, Yulan Meng, Xiaochun Ma

**Affiliations:** ^1^Department of Intensive Care Unit, The First Hospital of China Medical University, Nanjing Bei Street 155, Shenyang, Liaoning Province 110001, China; ^2^Department of Intensive Care Unit, Tacheng Hospital of China Medical University, Wenhua Road 22, Tacheng, Xinjiang Uygur Autonomous Region 834700, China

## Abstract

In sepsis-3, in contrast with sepsis-1, the definition “systemic inflammatory response” has been replaced with “dysregulated host response”, and “systemic inflammatory response syndrome” (SIRS) has been replaced with “sequential organ failure assessment” (SOFA). Although the definition of sepsis has changed, the debate regarding its nature is ongoing. What are the fundamental processes controlling sepsis-induced inflammation, immunosuppression, or organ failure? In this review, we discuss the heterogeneity of sepsis-3 and address the central role of inflammation in the pathogenesis of sepsis. An unbalanced pro- and anti-inflammatory response, inflammatory resolution disorder, and persistent inflammation play important roles in the acute and/or chronic phases of sepsis. Moreover, powerful links exist between inflammation and other host responses (such as the neuroendocrine response, coagulation, and immunosuppression). We suggest that a comprehensive evaluation of the role of the inflammatory response will improve our understanding of the heterogeneity of sepsis.

## 1. Introduction

The definition of sepsis was first proposed at an international conference in 1992 [1]. The conference emphasized that sepsis is an “ongoing process”. The terms systemic inflammatory response syndrome (SIRS), sepsis, severe sepsis, septic shock, and multiple organ dysfunction syndrome (MODS) began to be used in clinical practice [1]. Sepsis (sepsis-1) was defined as the identification of two or more SIRS criteria, in addition to a known or suspected infection. SIRS criteria included four clinical signs that, when altered, were thought to induce an inflammatory response: temperature, heart rate, respiratory rate, and white blood cell count [1]. In 2016, the Third International Consensus taskforce framed the definition of sepsis-3 and recommended that sepsis be defined as life-threatening organ dysfunction caused by the dysregulation of the host response due to an infection. Organ dysfunction can be identified as an acute alteration of the total sequential organ failure assessment (SOFA) score: at least two points higher than at the onset of the infection [2]. Moreover, to ensure early bedside evaluation of the likelihood of sepsis in a patient, a quick SOFA (qSOFA) score was introduced, which focuses on the following criteria: alteration of the mental status, systolic blood pressure (100 mm Hg or less), or respiratory rate (at least 22/min) [2].

### 1.1. Why the Change from Sepsis-1 to Sepsis-3?

The reasons for changing the definition from sepsis-1 to sepsis-3 included the following: an overwhelming focus on inflammation; a misleading continuum model; SIRS criteria lacking adequate sensitivity and specificity; and multiple definitions for sepsis, septic shock, and organ dysfunction that led to differences in the reported incidence and mortality [6, 7]. Moreover, the sepsis-3 definition deemphasizes SIRS in defining sepsis and focuses instead on organ dysfunction, because the complex pathobiology of sepsis includes both proinflammatory and anti-inflammatory responses. In addition, the SIRS score might not be accurate in critically ill patients with mechanical ventilation, sedation, and analgesia, which might affect the respiratory rate and heart rate scores.

Taken together, the prognostic accuracy for in-hospital mortality [8] of the sepsis-3 definition is higher than for sepsis-1. More extensively standardized quantification of organ dysfunction (through SOFA) criteria may improve spatial and temporal comparisons of sepsis cohorts.

### 1.2. The Weaknesses of Sepsis-3

Despite the strength of the new definition, it is yet to be universally embraced by the medical community. Several concerns regarding the new definition are outlined below. Firstly, the new definition, i.e., the shift in emphasis from SIRS to organ dysfunction, may delay early identification and treatment of sepsis [9, 10]. Secondly, distinguishing between infectious and noninfectious causes remains challenging.

**Figure 1:**
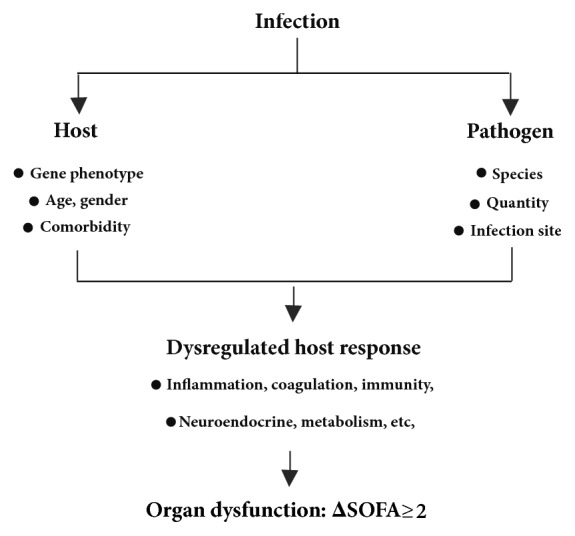
The heterogeneity of sepsis-3. Once sepsis-3 was defined, its heterogeneity became apparent. The response to infection depends on the host and the pathogen, and dysregulated host responses are heterogeneous and complicated. SOFA, sequential organ failure assessment.

Although the definition has changed, the debate on the nature of sepsis is ongoing. In our opinion, one of the biggest challenges for sepsis-3 is heterogeneity [10, 11]. It is well established that the response to infection depends on two factors: the host and the pathogen. Host factors include the genotype and phenotype; age (young versus old), chronic diseases (diabetes, hepatocirrhosis, kidney failure, cardiac disease, chronic obstructive pulmonary disease, etc.), and severe immunosuppression (long-term glucocorticoid therapy, tumor chemotherapy, human immunodeficiency virus infection, liver transplantation, etc.). Pathogen factors include the pathogen species (viral, bacterial, or fungal), the infection site (pneumonia, peritonitis, urinary infection, or meningitis), and others (Figure 1). Pooling heterogeneous infectious diseases and heterogeneous host responses under one umbrella (sepsis-3), and assuming that they all should be approached using the same new adjunctive therapy, might be counterproductive [10]. If hundreds of clinical studies based on the new sepsis criteria are performed, what will we learn? For example, the fluid resuscitation strategy for sepsis is quite different in patients with pneumonia and peritonitis, young and old patients, or patients with and without chronic cardiac failure. Clinicians in the intensive care unit (ICU) currently appear to be caught in a vicious cycle: single-center and small-sample studies usually yield encouraging positive results, while the results of large-sample, multicenter studies are typically disappointing.

**Figure 2:**
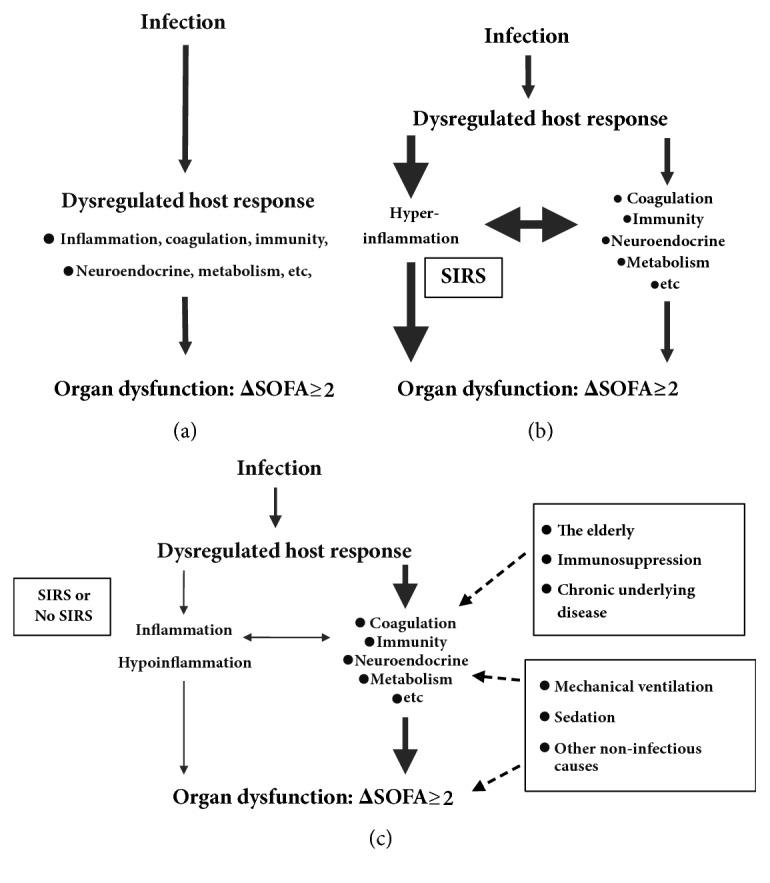
The heterogeneous inflammatory response in sepsis-3. According to the definition of sepsis-3, the dysregulated host response, as a bridge between the infection (the cause) and organ dysfunction (the outcome), is complicated and heterogeneous (a). In this review, we mainly discuss the central but heterogeneous role of the inflammatory response in sepsis. We propose a hypothetical classification of sepsis into “classical sepsis” (b) and “non-classical sepsis” (c). Hyperinflammation is regarded as the main cause of organ dysfunction in “classical sepsis” (b). Organ dysfunction might be caused by other host or noninfectious factors rather than hyperinflammation in “non-classical sepsis” (c). We believe that sepsis requires stratification and precise treatment. SIRS, systemic inflammatory response syndrome; SOFA, sequential organ failure assessment.

In addition to its heterogeneity, many clinicians are confused about the role of the inflammatory response in sepsis-3. What is the essence of sepsis? In contrast with sepsis-1, in sepsis-3, the “systemic inflammatory response” was replaced with “dysregulated host response”, and SIRS was changed to SOFA. The dysregulation of host responses is a complicated process and includes inflammation, the neuroendocrine response, coagulation, and metabolic responses. In fact, the neuroendocrine response and coagulation are closely linked to inflammation (Figure 2). In sepsis-3, it is more important to distinguish the interactions between the infection, inflammation, other host responses, and life-threatening organ dysfunction than in sepsis-1 (Figures 1 and 2). In younger patients without an underlying chronic disease, hyperinflammation and SIRS are still common and are regarded as the main causes of organ dysfunction in the early stage of sepsis. This is termed the “classical sepsis phenotype” (Figure 2(b)). In elderly patients with an underlying chronic disease, the infection might act as an initiating agent, and the inflammatory response might not be sufficiently intense to directly induce organ dysfunction. Other host or noninfectious factors, such as chronic underlying diseases (acute heart failure induced by pneumonia in a patient with chronic heart failure), mechanical ventilation, and sedation or general anesthetic (e.g., laparoscopic surgery for acute cholecystitis in a patient with chronic renal failure), might lead to or aggravate organ dysfunction** (**ΔSOFA ≥ 2, Figure 2(c)). Moreover, in very elderly patients, because of poor organ reserves, the infection often becomes “the straw that broke the camel's back” (Figure 2(c)). Although both groups of patients would be diagnosed with sepsis-3 based on their SOFA scores (ΔSOFA ≥ 2), the pathophysiological processes and treatment strategies would be vastly different for each group. We propose that the difference between the two types of sepsis is not limited to the age or comorbidity; indeed, it hinges on the role of inflammation in organ dysfunction.

During the acute and chronic phases of sepsis, an unbalanced inflammatory response to infection plays a key role in pathogenesis and organ dysfunction. We believe that an in-depth and comprehensive evaluation of the role of the inflammatory response will improve the understanding of sepsis-3, especially its heterogeneity.

## 2. The Relationship between Proinflammation and Anti-Inflammation in Sepsis

The immune response is usually divided into two components: innate and adaptive immunity. Generally, the acute inflammatory response is mainly associated with innate immunity, including neutrophils and macrophages. Adaptive immunity, which includes T-cells, B-cell, and dendritic cells, is associated with immunosuppression and secondary infection [12]. The activation of innate immunity involves pattern recognition receptors (PRRs) that recognize microbial components (pathogen-associated molecular patterns [PAMPs]) and biomolecules from damaged tissues (damage-associated molecular patterns [DAMPs]) [13]. PRRs are either membrane-bound or cytoplasmic, according to their orientation on or within the cell, and include toll-like receptors (TLRs), nucleotide-binding oligomerization domain- (NOD-) like receptors (NLRs), and C-type lectin receptors (CLRs) [14, 15].

### 2.1. Simultaneous Proinflammatory and Anti-Inflammatory Responses in Sepsis

During sepsis, the innate immune system activated by PAMPs and DAMPs releases multiple inflammatory cytokines in a process known as the “cytokine storm,” which results in a severe and persistent inflammatory response [16]. In addition, excessive inflammatory responses lead to cell and tissue damage that initiate organ dysfunction and even multiple organ failure [16].

**Figure 3:**
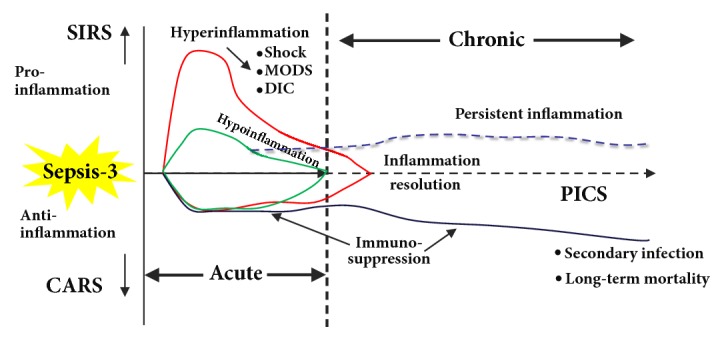
Two-phase model of sepsis-3. In the acute phase of sepsis, the host inflammatory response to an infection is heterogeneous, and sepsis may be classified as “classical” (hyperinflammation) and “non-classical” (hypoinflammation) (Figure 2). In the chronic phase of sepsis, persistent inflammation, immunosuppression, and catabolic syndrome (PICS) is the main cause of secondary infection and long-term mortality. CARS, compensatory anti-inflammatory response syndrome; DIC, disseminated intravascular coagulation; MODS, multiple organ dysfunction syndrome; SIRS, systemic inflammatory response syndrome; SOFA, sequential organ failure assessment. Adapted and modified from Hotchkiss* et al.* [3], Mira* et al.* [4], and Gentile* et al.* [5].

Formerly, the proinflammatory response was thought to drive early mortality in the first several days of sepsis, while the compensatory anti-inflammatory response was thought to induce organ failure, immune suppression, and mortality weeks later [17]. However, new insights from genomic analyses of tissue samples from septic patients [18] have confirmed a persistent and simultaneous inflammatory and anti-inflammatory state, driven by the dysfunction of the innate and adaptive immunity, which ultimately culminates in persistent organ injury and patient death (Figure 3) [16, 19, 20].

The roles of immune cell PRRs are only partially understood. TLR4 has become the focus of comprehensive research because of its role in the recognition of lipopolysaccharide (LPS). TLR4 plays a key role in the sepsis model, although its role in the regulation of inflammation varies with the cell type [21]. Interestingly, TLR4 induces not only proinflammatory reactions, but also anti-inflammatory reactions, illustrating the “balance” of the inflammatory response to LPS [22]. It has been shown that proinflammatory and anti-inflammatory responses are equally important and likely represent targets for future inflammation-based immunotherapy to improve the long-term outcomes of sepsis [3, 23–25].

Efforts have been dedicated to testing anti-TLR4 agents, such as eritoran, a myeloid differentiation protein (MD) 2-TLR4 antagonist, in patients with severe sepsis [26–28]. However, compared to the placebo, in a large randomized, double-blind, placebo-controlled, multi-national clinical trial, treatment with eritoran did not reduce the 28-d mortality of patients with severe sepsis [28]. This may be explained by the contributions of many other PRRs and PAMPs and/or DAMPs in driving the host response to sepsis. In addition, we believe that TLR4-specific blockers may be beneficial to patients with high levels of LPS in plasma. This also reflects the concept of precision therapy.

### 2.2. The Role of Inflammation Resolution in Sepsis

The inflammation resolution cascade was thought to be initiated several days after the primary sepsis episode had passed. However, Dalli* et al.* [29] discovered that resolvins, a group of specialized proresolving mediators (SPMs), are rapidly formed (within 4 h) during the initial phase of inflammation and improved the survival rates in mice infected with* Escherichia coli*.

The resolution of inflammation after sepsis is not a passive process of inhibiting cytokine production to alleviate the inflammatory response. Instead, evidence that it may be an active programmed response has emerged [30]. Effective resolution of the inflammatory response in tissues requires the simultaneous recruitment and differentiation of macrophages to terminate the recruitment of granulocytes. This facilitates the removal of inflammatory cells and tissue fragments, restoring tissue homeostasis [31]. Hence, it is reasonable to hypothesize that the persistent inflammation in patients following sepsis might, at least in part, be caused by the dysfunctional resolution of inflammation (Figure 3) [32].

Historically, therapeutic interventions aimed at inhibiting the acute inflammatory response to infection and subsequent sepsis using glucocorticoids, nonsteroidal anti-inflammatory agents, and antitumor necrosis factor- (TNF-) *α* antibodies have repeatedly failed to improve the outcomes in patients with sepsis and septic shock [33]. Therefore, many scholars have questioned the value of anti-inflammatory therapy in sepsis. However, in contrast with the traditional broad-spectrum or single-targeted anti-inflammatory and immunosuppressive agents, SPMs are endogenously driven from native processes and promote the inhibition of the inflammatory response while allowing for full clearance of the bacterial infection [34]. In animal models, the augmentation of resolution also seems to decrease the required dose of antibiotics for bacterial infection clearance [35, 36]. Based on the above, SPMs are expected to be useful to evaluate the need for inflammatory regulation therapy in sepsis.

### 2.3. Lessons from the Failure of Treatments Targeting Inflammation in Sepsis

Although growing evidence suggests that cytokines play a key pathophysiological role in the infection process, no specific inflammation-targeting therapies were shown to be effective against sepsis. Thus, the notion of SIRS as the essence of sepsis is questionable and is also considered to be one of the reasons for the updated definition of sepsis.

The failure of inflammation-targeting therapies in sepsis could have several explanations.

Firstly, septic patients are heterogeneous. As discussed above, the manifestations of sepsis are varied, including different sources of infection, different mechanisms of development, and different degrees of inflammation, all leading to different responses to treatment (Figure 1) [33, 37]. Using the same treatment strategy for heterogeneous cases of sepsis will not exert any beneficial effects. Patients included in sepsis studies should be selected based on specific inflammatory parameters or biomarkers. For example, patients with high levels of proinflammatory cytokines might respond to an anti-inflammatory treatment [33, 37].

Secondly, because hundreds of signaling factors are involved in the inflammation response in sepsis, therapeutic strategies targeting specific cytokines will be largely ineffective in treating sepsis [28, 38].

Thirdly, each cytokine generated during sepsis is a double-edged sword. On the one hand, proinflammatory cytokines cause damage; on the other hand, the host needs them to remove pathogenic microorganisms. For example, TLR4 inhibition plays different roles in the mild or severe cecal ligation perforation (CLP) model of sepsis [39, 40]. TLR4 plays an essential role in host defense against low-grade polymicrobial sepsis by mediating the migratory and/or phagocytic functions of neutrophils, attenuating inflammation, reducing the generation of reactive oxygen species (ROS), and enhancing bacterial clearance [39]. However, low bacteremia and a high survival rate in TLR4-deficient mice with lethal polymicrobial sepsis were reported in another study, indicating the harmful role of TLR4 signaling in severe polymicrobial sepsis [40].

Finally, a model of concurrent hyperinflammation and immunosuppression has been proposed to explain the complex pathogenesis of sepsis at various time points (Figure 3) [3, 4].

Hyperinflammation-induced organ failure is thought to be the most common cause of death during the first days of sepsis. In the chronic phase of sepsis, persistent inflammation, immunosuppression, and catabolic syndrome (PICS) becomes the main cause of secondary ICU-acquired infections and long-term mortality [4] (Figure 3). However, it is difficult to distinguish hyperinflammation and immunosuppression in patients with sepsis. Therefore, the timing and course of anti-inflammatory treatments also require discussion. Assessing the inflammatory and immune status of sepsis calls for a precise stage-dependent therapy [41].

## 3. The Relationship between the Inflammatory Response and the Dysregulated Host Response in Sepsis

### 3.1. The Neural Control of Inflammation in Sepsis

Many bacterial toxins (e.g., LPS) or host mediators (e.g., interleukin- [IL-] 1 and TNF) can interact directly with sensory neurons to mediate the generation of afferent action potentials [42]. These afferent signals arise in the sensory vagus nerve and terminate in the nucleus tractus solitarii in the brainstem. They are required to induce fever, sickness behavior, and other neurological inflammatory responses [42].

Catecholamines are the main transmitters of the sympathetic branch of the autonomic nervous system (ANS). They act by binding to adrenergic receptors. In the early phase of sepsis, the levels of circulating catecholamines are high, which potentiates the initial inflammatory response. Catecholamines exert immunomodulatory effects via adrenergic receptors expressed by immune cells [43–45]. The release of proinflammatory cytokines by neutrophils and macrophages particularly underlies the adrenergic regulation by catecholamines [46, 47].

Meanwhile, previous studies demonstrated that a neural reflex, the “inflammatory reflex,” modulates regional and systemic inflammation in sepsis [42]. In experimental sepsis, activation of the inflammatory reflex by selective alpha 7-nicotinic acetylcholine receptor (*α*7-nAChR) agonists or by direct electrical stimulation of the vagus nerve decreases not only proinflammatory cytokine generation, but also the survival rate [48–50]. Similarly, electrical stimulation of the vagus nerve inhibited TNF synthesis in wild-type mice but failed to inhibit TNF synthesis in alpha 7-deficient mice [51]. The association between the changes in vagus nerve activity and systemic inflammatory responses has been observed. Heart rate variability, an indicator of vagus nerve activity, was analyzed in earlier studies. These studies revealed that the vagus nerve signals change before severe sepsis, which might be associated with compromised cardiac function [52–55]. Consistent with these observations, early changes in the heart rate variability recorded at admission to the emergency department have been associated with an increased risk of developing septic shock and death [53, 56, 57]. Thus, exploiting the neural control mechanisms of inflammation provides a new and exciting possibility for the treatment of dysregulated inflammation in sepsis.

### 3.2. The Interaction between Inflammation and Endocrine Dysfunction in Sepsis

Endocrine dysfunction in sepsis is characterized by an altered hormonal profile that leads to reduced adrenal responsiveness to the adrenocorticotropic hormone (ACTH), hyperglycemia, euthyroid sick syndrome, and others [58].

The hypothalamic-pituitary-adrenal axis (HPA) is the main neuroendocrine structure that regulates the adaptive response to different stressors. It is based on the interconnection of the sympathoadrenal and neurohypophyseal systems, which in turn are responsible for catecholamine secretion, vasopressin release, and cytokine activation. Dysfunction of the HPA in sepsis is caused by complex and multifactorial mechanisms and results in either an absolute deficiency of serum hormone levels (reduced production of the corticotropin-releasing hormone, ACTH, and cortisol) or a relative hormonal deficiency caused by a dysfunction of their receptors [58, 59]. It has been established that adrenal insufficiency in sepsis (especially septic shock) is in part responsible for the reduction of vascular reactivity to vasopressors and is associated with an increased risk of death [60]. Long course and low dose (e.g., IV hydrocortisone < 400 mg/day for ≥ 3 days at full dose) corticosteroids are recommended in patients with septic shock who are not responsive to fluids and moderate- to high-dose vasopressor therapy (> 0.1 *μ*g/kg/min norepinephrine, or equivalent) [61]. More recently, the administration of hydrocortisone and fludrocortisone was reported to significantly reduce 90-day mortality in patients with septic shock [62]. However, in ADRENAL Trial, hydrocortisone (200 mg/day for 7 days) did not improve the prognosis of patients with septic shock undergoing mechanical ventilation [63]. It is worth noting that chronic corticosteroid use increases the risk of infection due to long-term immunosuppression [64]. Corticosteroid administration is not recommended in adult patients with sepsis without shock [61].

Hyperglycemia and insulin resistance are common characteristics of patients with sepsis. Various mechanisms are involved in hyperglycemia, such as the activity of circulating counter-regulatory hormones, cytokine-related insulin resistance, activation of the hepatic glycogenolysis and gluconeogenesis, hepatic clearance of serum lactate in the Cori cycle, and medications (e.g., glucocorticoids) [59, 65, 66]. The mechanisms by which hyperglycemia increases morbidity and mortality in sepsis include abnormalities of the host response (especially an elevated generation of proinflammatory cytokines), ROS formation, reduced chemotaxis and phagocytosis, prothrombotic effects, and endothelial cell injury [59, 65, 67].

Thyroid dysfunction observed in sepsis is considered to constitute a part of the adaptive metabolic response, as an attempt to increase resistance to different stressors by reducing cellular metabolic activity [68]. The overall levels of thyroxine 4, free thyroxine 4, and thyroid-stimulating hormone (TSH) may also decrease with increasing severity of the illness [69]. Moreover, decreased baseline thyroid function might be associated with a worse outcome for patients with sepsis or septic shock [68]. Dysfunction of the hypothalamic-pituitary-thyroid axis in sepsis could be attributed to a variety of cytokines [69]. Several inflammatory cytokines (e.g., IL-1*β* and IL-6) can directly or indirectly inhibit thyroid function at different levels, thereby reducing the secretion of the hypothalamic thyrotropin-releasing hormone (TRH) or directly inhibiting the release of TSH [65, 68].

Taken together, hyperinflammation can lead to endocrine and metabolic dysfunction and can exacerbate inflammation and immune disorders in sepsis. Conversely, a patient with a chronic endocrine and metabolic disease who is suffering from infection might present different clinical features.

### 3.3. The Crosstalk between Inflammation and Coagulation in Sepsis

Sepsis is frequently complicated by coagulopathy and disseminated intravascular coagulation (DIC), which is a strong predictor of mortality [70–72]. In the initial phase of DIC, thrombin activation leads to fibrin formation (hypercoagulability); this is followed by the consumption of coagulation factors and thrombocytopenia (hypocoagulability) [73]. In the late phase of DIC, microvascular fibrin deposition and microthrombosis are often associated with the development of multiple organ failure [73].

Inflammation and coagulation play a pivotal role in the pathogenesis of sepsis [74]. Because of the extensive crosstalk between inflammation and coagulation in sepsis [74–77], we believe that a detailed analysis of the changes in blood coagulation function can provide multiple clues to distinguish whether organ dysfunction or shock is caused by hyperinflammation. Only platelet counting is used to evaluate the coagulation function in the SOFA score, which is obviously not sufficient. Examination of DIC scores at the beginning of DIC treatment revealed a greater treatment efficacy in pre-DIC patients than in DIC patients [78]. The outcome worsens as the DIC score increases, suggesting the importance of both early diagnosis and treatment of DIC. The levels of D-dimer and fibrin/fibrinogen degradation products (FDP) were significantly lower in patients with pre-DIC than in those with DIC, whereas there were no significant differences in the levels of thrombin-antithrombin complex (TAT), antithrombin (AT), and thrombomodulin (TM) [79]. In contrast with the low level of fibrinogen in traumatic coagulopathy, in the early phase of sepsis, the level of fibrinogen is usually normal or high. The diagnostic criteria of the Japanese Association for Acute Medicine (JAAM) for acute-stage DIC were initially created using fibrinogen for DIC diagnosis at ≥ 5 points; however, in practice, fibrinogen did not have any diagnostic significance. The criteria were hence modified by eliminating fibrinogen for DIC diagnosis at ≥ 4 points [80]. Similarly, in an ongoing randomized controlled trial of heparin therapy for sepsis DIC (NCT02654561), we used the modified ISTH score (no fibrinogen score). Of course, DIC is also characterized by some heterogeneity [81]; this heterogeneity should be distinguished and interpreted in a clinical setting.

## 4. The Relationship between the Inflammatory Response and Immunosuppression in Sepsis

At present, immunosuppression in sepsis is a major focus of clinical research. Indeed, extensive studies have revealed that patients in the late phase of sepsis are highly susceptible to subsequent infections by multiple resistant bacteria or fungi [82, 83]. Although modern medical strategies have improved the short-term outcome of sepsis patients, they also lead to a more persistent disease state, which turns into an immunosuppressive phenotype resulting in an increased incidence of delayed death. Hotchkiss* et al.* showed that defective responses in the innate and adaptive immune systems are a hallmark of immunosuppression during sepsis [84–86]. Major mechanisms of sepsis-induced immunosuppression include (a) apoptosis of immune cells; (b) compromised T-cell effector function, T-cell exhaustion, and impaired antigen presentation; and (c) endotoxin tolerance or impaired cytokine responses [19, 87–89]. In fact, the impairment of the immune system is at least partially caused by inflammation.

### 4.1. Apoptosis of the Immune Cells

Apoptosis of lymphocytes and antigen-presenting cells (dendritic cells, T-cells, and B-cells) is considered a hallmark of septic immune suppression [90, 91]. In fact, lymphopenia occurring 4 d after the onset of sepsis is associated with the development of secondary infection and is predictive of long-term mortality 1 year after sepsis [92].

During sepsis, a large number of inducers are generated and released, including cytokines such as TNF-*α*, high mobility group box-1 protein (HMGB1), oxygen-free radicals, and nitric oxide (NO) [93]. The well-known mechanisms of sepsis-induced lymphocyte apoptosis involve both the receptor-caspase-8-mediated (extrinsic) and the mitochondrial caspase-9-mediated (intrinsic) pathways of apoptosis [93, 94]. Apoptosis has shown promise as a new target for sepsis treatment in the animal models. PD-1 was found to mediate T-cell apoptosis, and anti-PD-1 antibody prevented sepsis-induced lymphocyte depletion and decreased mortality in a mice CLP model [95, 96]. Furthermore, other immunomodulatory agents, such as IL-7, IL-15, and IL-33, have improved lymphocyte apoptosis and survival in septic models [97].

### 4.2. Endotoxin Tolerance

Monocytes from septic patients are typically characterized by a diminished capacity to release proinflammatory cytokines such as TNF-*α*, IL-1, IL-6, and IL-12; on the other hand, the release of anti-inflammatory mediators, such as IL-1 receptor antagonist and IL-10, is either unimpaired or enhanced [88, 98]. In clinical studies, the magnitude and persistent nature of endotoxin tolerance is associated with increased mortality and nosocomial infections [99, 100]. Endotoxin tolerance, caused by the first inflammation hit, is considered to be a clinical feature of septic immune suppression. Conversely, persistent endotoxin tolerance in sepsis patients might be caused by persistent inflammation, occult or residual infection, or endotoxins from the gut flora.

Thus, an early, timely, and effective anti-infection treatment, reduction of the hyperinflammation response in the early phase of sepsis, and inhibition of the persistent inflammatory response in the chronic phase of sepsis continue to be promising strategies for addressing sepsis-induced immunosuppression (Figure 3).

Interestingly, a recent study reported a sepsis patient who acquired a secondary infection during their ICU stay and displayed profound hyperinflammatory responses, in addition to various immunosuppressive characteristics [101]. Thus, immunosuppression does not equate to a weak inflammatory response. In fact, not only the immune cells, but also other cells, including endothelial cells and platelets, can produce inflammatory cytokines. The concurrence of hyperinflammation and immunosuppression indicate that the role of inflammation in sepsis is probably much more complicated than we think.

## 5. Conclusions 

Even in the era of sepsis-3, the inflammatory response plays an extremely important role in the pathophysiology of sepsis. It includes hyperinflammation during the early phase of sepsis, defective inflammatory resolution, and persistent inflammation in the chronic phase of sepsis. A better understanding of the balance of proinflammation and anti-inflammation and the interconnection between inflammation and other host responses (e.g., the neuroendocrine response, coagulation, and immunosuppression) will facilitate the analysis and evaluation of the relationship between infection, inflammation, and organ dysfunction. This will also help ICU physicians in understanding the heterogeneity of sepsis.

## Additional Points

*Highlights.* (i) The heterogeneity of sepsis-3 is discussed in this review. (ii) The central role of inflammation in the pathogenesis of sepsis-3 is considered. (iii) Powerful links exist between inflammation and other host responses in sepsis-3. (iv) Understanding the inflammatory response will improve our understanding of sepsis-3.

## References

[1] Bone R. C., Balk R. A., Cerra F. B. (2009). Definitions for sepsis and organ failure and guidelines for the use of innovative therapies in sepsis: the ACCP/SCCM Consensus Conference Committee. *CHEST*.

[2] Singer M., Deutschman C. S., Seymour C. W. (2016). The third international consensus definitions for sepsis and septic shock (sepsis-3). *The Journal of the American Medical Association*.

[3] Hotchkiss R. S., Monneret G., Payen D. (2013). Sepsis-induced immunosuppression: from cellular dysfunctions to immunotherapy. *Nature Reviews Immunology*.

[4] Mira J. C., Gentile L. F., Mathias B. J. (2016). Sepsis pathophysiology, chronic critical illness, and persistent inflammation-immunosuppression and catabolism syndrome. *Critical Care Medicine*.

[5] Gentile L. F., Cuenca A. G., Efron P. A. (2012). Persistent inflammation and immunosuppression: a common syndrome and new horizon for surgical intensive care. *Journal of Trauma and Acute Care Surgery*.

[6] Peach B. C. (2017). Implications of the new sepsis definition on research and practice. *Journal of Critical Care*.

[7] Rhee C., Klompas M. (2017). New Sepsis and Septic Shock Definitions: Clinical Implications and Controversies. *Infectious Disease Clinics of North America*.

[8] Raith E. P., Udy A. A., Bailey M. (2017). Prognostic accuracy of the SOFA score, SIRS criteria, and qSOFA score for in-hospital mortality among adults with suspected infection admitted to the intensive care unit. *Journal of the American Medical Association*.

[9] Simpson S. Q. (2016). New sepsis criteria A change we should not make. *CHEST*.

[10] Cortes-Puch I., Hartog C. S. (2016). Change is not necessarily progress: Revision of the sepsis definition should be based on new scientific insights. *American Journal of Respiratory and Critical Care Medicine*.

[11] Deutschman C. S. (2016). Imprecise medicine: The limitations of sepsis-3. *Critical Care Medicine*.

[12] Bhan C., Dipankar P., Chakraborty P., Sarangi P. P. (2016). Role of cellular events in the pathophysiology of sepsis. *Inflammation Research*.

[13] Medzhitov R., Janeway C. J. (2000). Advances in immunology: innate immunity. *The New England Journal of Medicine*.

[14] Iwasaki A., Medzhitov R. (2010). Regulation of adaptive immunity by the innate immune system. *Science*.

[15] Broz P., Monack D. M. (2013). Newly described pattern recognition receptors team up against intracellular pathogens. *Nature Reviews Immunology*.

[16] Rittirsch D., Flierl M. A., Ward P. A. (2008). Harmful molecular mechanisms in sepsis. *Nature Reviews Immunology*.

[17] Bone R. C., Grodzin C. J., Balk R. A. (1997). Sepsis: a new hypothesis for pathogenesis of the disease process. *CHEST*.

[18] Boomer J. S., To K., Chang K. C. (2011). Immunosuppression in patients who die of sepsis and multiple organ failure. *Journal of the American Medical Association*.

[19] Xiao W., Mindrinos M. N., Seok J. (2011). A genomic storm in critically injured humans. *The Journal of Experimental Medicine*.

[20] Cuenca A. G., Gentile L. F., Lopez M. C. (2013). Development of a genomic metric that can be rapidly used to predict clinical outcome in severely injured trauma patients. *Critical Care Medicine*.

[21] Deng M., Scott M. J., Loughran P. (2013). Lipopolysaccharide clearance, bacterial clearance, and systemic inflammatory responses are regulated by cell type-specific functions of tlr4 during sepsis. *The Journal of Immunology*.

[22] Waltz P., Carchman E. H., Young A. C. (2011). Lipopolysaccaride induces autophagic signaling in macrophages via a TLR4, heme oxygenase-1 dependent pathway. *Autophagy*.

[23] Delano M. J., Moldawer L. L. (2009). Magic bullets and surrogate biomarkers circa 2009. *Critical Care Medicine*.

[24] Bosmann M., Ward P. A. (2013). The inflammatory response in sepsis. *Trends in Immunology*.

[25] Hutchins N. A., Unsinger J., Hotchkiss R. S., Ayala A. (2014). The new normal: immunomodulatory agents against sepsis immune suppression. *Trends in Molecular Medicine*.

[26] Rossignol D. P., Wasan K. M., Choo E. (2004). Safety, pharmacokinetics, pharmacodynamics, and plasma lipoprotein distribution of eritoran (E5564) during continuous intravenous infusion into healthy volunteers. *Antimicrobial Agents and Chemotherapy*.

[27] Barochia A., Solomon S., Cui X., Natanson C., Eichacker P. Q. (2011). Eritoran tetrasodium (E5564) treatment for sepsis: review of preclinical and clinical studies. *Expert Opinion on Drug Metabolism & Toxicology*.

[28] Opal S. M., Laterre P.-F., Francois B. (2013). Effect of eritoran, an antagonist of MD2-TLR4, on mortality in patients with severe sepsis: The ACCESS randomized trial. *The Journal of the American Medical Association*.

[29] Dalli J., Chiang N., Serhan C. N. (2015). Elucidation of novel 13-series resolvins that increase with atorvastatin and clear infections. *Nature Medicine*.

[30] Serhan C. N., Clish C. B., Brannon J., Colgan S. P., Chiang N., Gronert K. (2000). Novel functional sets of lipid-derived mediators with antiinflammatory actions generated from omega-3 fatty acids via cyclooxygenase 2-nonsteroidal antiinflammatory drugs and transcellular processing. *The Journal of Experimental Medicine*.

[31] Buckley C. D., Gilroy D. W., Serhan C. N. (2014). Proresolving lipid mediators and mechanisms in the resolution of acute inflammation. *Immunity*.

[32] Buechler C., Pohl R., Aslanidis C. (2017). Pro-resolving molecules—New approaches to treat sepsis?. *International Journal of Molecular Sciences*.

[33] Marshall J. C. (2014). Why have clinical trials in sepsis failed?. *Trends in Molecular Medicine*.

[34] Serhan C. N. (2017). Treating inflammation and infection in the 21st century: New hints from decoding resolution mediators and mechanisms. *The FASEB Journal*.

[35] Chiang N., Fredman G., Bäckhed F. (2012). Infection regulates pro-resolving mediators that lower antibiotic requirements. *Nature*.

[36] Ueda T., Fukunaga K., Seki H. (2014). Combination therapy of 15-epi-lipoxin a4 with antibiotics protects mice from escherichia coli-induced sepsis. *Critical Care Medicine*.

[37] Chousterman B. G., Swirski F. K., Weber G. F. (2017). Cytokine storm and sepsis disease pathogenesis. *Seminars in Immunopathology*.

[38] Reinhart K., Wiegand-Löhnert C., Grimminger F. (1996). Assessment of the safety and efficacy of the monoclonal anti-tumor necrosis factor antibody-fragment, MAK 195F, in patients with sepsis and septic shock: A multicenter, randomized, placebo-controlled, dose-ranging study. *Critical Care Medicine*.

[39] Zhang M., Zou L., Feng Y. (2014). Toll-like receptor 4 is essential to preserving cardiac function and survival in low-grade polymicrobial sepsis. *Anesthesiology*.

[40] Alves-Filho J. C., De Freitas A., Russo M., Cunha F. Q. (2006). Toll-like receptor 4 signaling leads to neutrophil migration impairment in polymicrobial sepsis. *Critical Care Medicine*.

[41] Bermejo-Martin J. F., Andaluz-Ojeda D., Almansa R. (2016). Defining immunological dysfunction in sepsis: A requisite tool for precision medicine. *Infection*.

[42] Deutschman C. S., Tracey K. J. (2014). Sepsis: current dogma and new perspectives. *Immunity*.

[43] Bergmann M., Sautner T. (2002). Immunomodulatory effects of vasoactive catecholamines. *Wien Klin Wochenschr*.

[44] Oberbeck R. (2006). Catecholamines: Physiological immunomodulators during health and illness. *Current Medicinal Chemistry*.

[45] Oberbeck R., Schmitz D., Wilsenack K. (2004). Adrenergic modulation of survival and cellular immune functions during polymicrobial sepsis. *Neuroimmunomodulation*.

[46] Spengler R. N., Allen R. M., Remick D. G., Strieter R. M., Kunkel S. L. (1990). Stimulation of *α*-adrenergic receptor augments the production of macrophage-derived tumor necrosis factor. *The Journal of Immunology*.

[47] Flierl M. A., Rittirsch D., Nadeau B. A. (2007). Phagocyte-derived catecholamines enhance acute inflammatory injury. *Nature*.

[48] Huston J. M., Gallowitsch-Puerta M., Ochani M. (2007). Transcutaneous vagus nerve stimulation reduces serum high mobility group box 1 levels and improves survival in murine sepsis. *Critical Care Medicine*.

[49] Pavlov V. A., Ochani M., Yang L. (2007). Selective *α*7-nicotinic acetylcholine receptor agonist GTS-21 improves survival in murine endotoxemia and severe sepsis. *Critical Care Medicine*.

[50] Wang H., Liao H., Ochani M. (2004). Cholinergic agonists inhibit HMGB1 release and improve survival in experimental sepsis. *Nature Medicine*.

[51] Wang H., Yu M., Ochani M. (2003). Nicotinic acetylcholine receptor *α*7 subunit is an essential regulator of inflammation. *Nature*.

[52] Thayer J. F., Lane R. D. (2009). Claude Bernard and the heart-brain connection: further elaboration of a model of neurovisceral integration. *Neuroscience & Biobehavioral Reviews*.

[53] Buchan C. A., Bravi A., Seely A. J. E. (2012). Variability analysis and the diagnosis, management, and treatment of sepsis. *Current Infectious Disease Reports*.

[54] Frasure-Smith N., Lespérance F., Irwin M. R., Talajic M., Pollock B. G. (2009). The relationships among heart rate variability, inflammatory markers and depression in coronary heart disease patients. *Brain, Behavior, and Immunity*.

[55] Werdan K., Schmidt H., Ebelt H. (2009). Impaired regulation of cardiac function in sepsis, SIRS, and MODS. *Canadian Journal of Physiology and Pharmacology*.

[56] Barnaby D., Ferrick K., Kaplan D. T., Shah S., Bijur P., Gallagher E. J. (2002). Heart rate variability in emergency department patients with sepsis. *Academic Emergency Medicine*.

[57] Chen W.-L., Kuo C.-D. (2007). Characteristics of Heart Rate Variability Can Predict Impending Septic Shock in Emergency Department Patients with Sepsis. *Academic Emergency Medicine*.

[58] Gheorghiţă V., Barbu A. E., Gheorghiu M. L. (2015). Endocrine dysfunction in sepsis: a beneficial or deleterious host response?. *GERMS*.

[59] Prigent H., Maxime V., Annane D. (2004). Clinical review: Corticotherapy in sepsis. *Critical Care*.

[60] Annane D., Sébille V., Troché G., Raphaël J.-C., Gajdos P., Bellissant E. (2000). A 3-level prognostic classification in septic shock based on cortisol levels and cortisol response to corticotropin. *Journal of the American Medical Association*.

[61] Annane D., Pastores S. M., Rochwerg B. (2017). Guidelines for the Diagnosis and Management of Critical Illness-Related Corticosteroid Insufficiency (CIRCI) in Critically Ill Patients (Part I): Society of Critical Care Medicine (SCCM) and European Society of Intensive Care Medicine (ESICM) 2017. *Critical Care Medicine*.

[62] Annane D., Renault A., Brun-Buisson C. (2018). Hydrocortisone plus Fludrocortisone for Adults with Septic Shock. *The New England Journal of Medicine*.

[63] Venkatesh B., Finfer S., Cohen J. (2018). Adjunctive Glucocorticoid Therapy in Patients with Septic Shock. *The New England Journal of Medicine*.

[64] Chaudhary N. S., Donnelly J. P., Moore J. X., Baddley J. W., Safford M. M., Wang H. E. (2017). Association of baseline steroid use with long-term rates of infection and sepsis in the REGARDS cohort. *Critical Care*.

[65] Khardori R., Castillo D. (2012). Endocrine and metabolic changes during sepsis: an update. *Medical Clinics of North America*.

[66] Levy B. (2006). Lactate and shock state: The metabolic view. *Current Opinion in Critical Care*.

[67] Koh G. C. K. W., Peacock S. J., van der Poll T., Wiersinga W. J. (2012). The impact of diabetes on the pathogenesis of sepsis. *European Journal of Clinical Microbiology & Infectious Diseases*.

[68] Angelousi A. G., Karageorgopoulos D. E., Kapaskelis A. M., Falagas M. E. (2011). Association between thyroid function tests at baseline and the outcome of patients with sepsis or septic shock: A systematic review. *European Journal of Endocrinology*.

[69] Fliers E., Kalsbeek A., Boelen A. (2014). Mechanisms in endocrinology: beyond the fixed setpoint of the hypothalamus-pituitary-thyroid axis. *European Journal of Endocrinology*.

[70] Levi M., Ten Cate H. (1999). Disseminated intravascular coagulation. *The New England Journal of Medicine*.

[71] Bakhtiari K., Meijers J. C. M., De Jonge E., Levi M. (2004). Prospective validation of the International Society of Thrombosis and Haemostasis scoring system for disseminated intravascular coagulation. *Critical Care Medicine*.

[72] Dhainaut J.-F., Yan S. B., Joyce D. E. (2004). Treatment effects of drotrecogin alfa (activated) in patients with severe sepsis with or without overt disseminated intravascular coagulation. *Journal of Thrombosis and Haemostasis*.

[73] Abraham E. (2000). Coagulation abnormalities in acute lung injury and sepsis. *American Journal of Respiratory Cell and Molecular Biology*.

[74] Levi M., van der Poll T. (2010). Inflammation and coagulation. *Critical Care Medicine*.

[75] Stouthard J. M. L., Levi M., Hack C. E. (1996). Interleukin-6 stimulates coagulation, not fibrinolysis, in humans. *Thrombosis and Haemostasis*.

[76] Schouten M., Wiersinga W. J., Levi M., van der Poll T. (2008). Inflammation, endothelium, and coagulation in sepsis. *Journal of Leukocyte Biology*.

[77] Bernard G. R., Vincent J.-L., Laterre P.-F. (2001). Efficacy and safety of recombinant human activated protein C for severe sepsis. *The New England Journal of Medicine*.

[78] Wada H., Wakita Y., Nakase T. (1995). Outcome of disseminated intravascular coagulation in relation to the score when treatment was begun. *Thrombosis and Haemostasis*.

[79] Okamoto K., Wada H., Hatada T. (2010). Frequency and hemostatic abnormalities in pre-DIC patients. *Thrombosis Research*.

[80] Lu B., Nakamura T., Inouye K. (2012). Novel role of PKR in inflammasome activation and HMGB1 release. *Nature*.

[81] Asakura H., Takahashi H., Uchiyama T. (2016). Proposal for new diagnostic criteria for DIC from the Japanese Society on Thrombosis and Hemostasis. *Thrombosis Journal*.

[82] Monneret G., Venet F., Kullberg B.-J., Netea M. G. (2011). ICU-acquired immunosuppression and the risk for secondary fungal infections. *Medical Mycology*.

[83] Otto G. P., Sossdorf M., Claus R. A. (2011). The late phase of sepsis is characterized by an increased microbiological burden and death rate. *Critical Care*.

[84] Hotchkiss R. S., Opal S. (2010). Immunotherapy for sepsis - A new approach against an ancient foe. *The New England Journal of Medicine*.

[85] Hotchkiss R. S., Coopersmith C. M., McDunn J. E., Ferguson T. A. (2009). The sepsis seesaw: tilting toward immunosuppression. *Nature Medicine*.

[86] Hotchkiss R. S., Monneret G., Payen D. (2013). Immunosuppression in sepsis: a novel understanding of the disorder and a new therapeutic approach. *The Lancet Infectious Diseases*.

[87] Boomer J. S., Green J. M., Hotchkiss R. S. (2014). The changing immune system in sepsis: is individualized immuno-modulatory therapy the answer?. *Virulence*.

[88] Biswas S. K., Lopez-Collazo E. (2009). Endotoxin tolerance: new mechanisms, molecules and clinical significance. *Trends in Immunology*.

[89] Patil N. K., Bohannon J. K., Sherwood E. R. (2016). Immunotherapy: A promising approach to reverse sepsis-induced immunosuppression. *Pharmacological Research*.

[90] Hotchkiss R. S., Tinsley K. W., Swanson P. E. (2002). Depletion of dendritic cells, but not macrophages, in patients with sepsis. *The Journal of Immunology*.

[91] Hotchkiss R. S., Swanson P. E., Freeman B. D. (1999). Apoptotic cell death in patients with sepsis, shock, and multiple organ dysfunction. *Critical Care Medicine*.

[92] Drewry A., Samra N., Skrupky L., Fuller B., Compton S., Hotchkiss R. (2014). Persistent lymphopenia after diagnosis of sepsis predicts mortality. *Shock*.

[93] Luan Y.-Y., Yao Y.-M., Xiao X.-Z., Sheng Z.-Y. (2015). Insights into the apoptotic death of immune cells in sepsis. *Journal of Interferon & Cytokine Research*.

[94] Hotchkiss R. S., Nicholson D. W. (2006). Apoptosis and caspases regulate death and inflammation in sepsis. *Nature Reviews Immunology*.

[95] Brahmamdam P., Inoue S., Unsinger J., Chang K. C., McDunn J. E., Hotchkiss R. S. (2010). Delayed administration of anti-PD-1 antibody reverses immune dysfunction and improves survival during sepsis. *Journal of Leukocyte Biology*.

[96] Zhang Y., Zhou Y., Lou J. (2010). PD-L1 blockade improves survival in experimental sepsis by inhibiting lymphocyte apoptosis and reversing monocyte dysfunction. *Critical Care*.

[97] Girardot T., Rimmelé T., Venet F., Monneret G. (2017). Apoptosis-induced lymphopenia in sepsis and other severe injuries. *Apoptosis*.

[98] Cavaillon J.-M., Adib-Conquy M. (2006). Bench-to-bedside review: endotoxin tolerance as a model of leukocyte reprogramming in sepsis. *Critical Care*.

[99] Monneret G., Lepape A., Voirin N. (2006). Persisting low monocyte human leukocyte antigen-DR expression predicts mortality in septic shock. *Intensive Care Medicine*.

[100] Hoogendijk A. J., Garcia-Laorden M. I., van Vught L. A. (2017). Sepsis patients display a reduced capacity to activate nuclear factor-*κ*B in multiple cell types. *Critical Care Medicine*.

[101] Van Vught L. A., Wiewel M. A., Hoogendijk A. J. (2017). The host response in patients with sepsis developing intensive care unit-acquired secondary infections. *American Journal of Respiratory and Critical Care Medicine*.

